# Supplementary material for: Yiai Fuzheng decoction inhibits triple-negative breast cancer by remodeling the immune microenvironment
Source: Front Immunol. 2025 Sep 30;16:1615631. doi: 10.3389/fimmu.2025.1615631 (PMC12518410; doi:10.3389/fimmu.2025.1615631)
Supplement: Supplementary file 2 [file DataSheet1.zip › Supplementary File 2/Treatment/C_vs_D/07_Pathway_Analysis/Enrichment_Analysis/smpdb_pathway/Enrichment_Barplot.pdf]

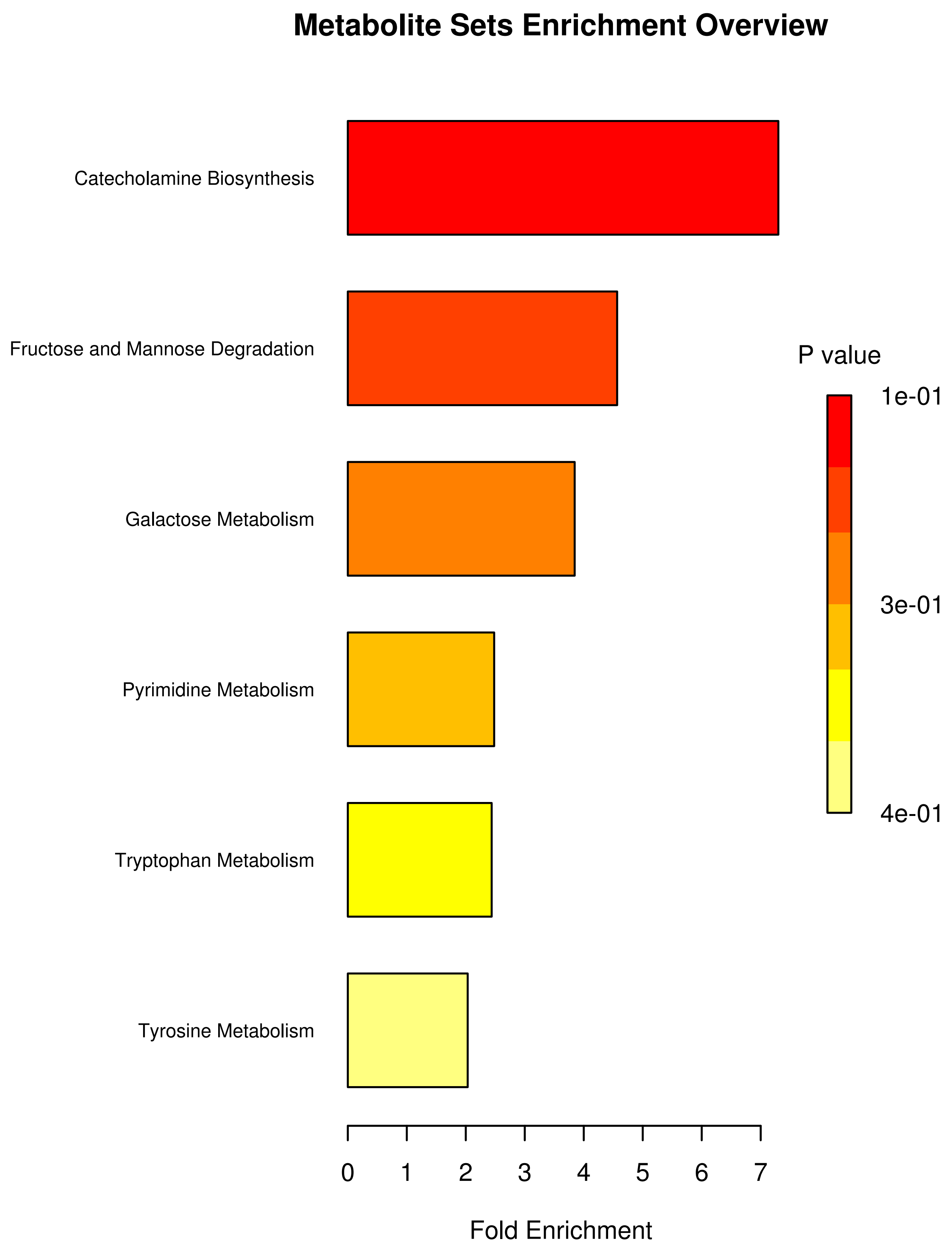
Metabolite Sets Enrichment Overview
Catecholamine Biosynthesis
Fructose and Mannose Degradation
Galactose Metabolism
Pyrimidine Metabolism
Tryptophan Metabolism
Tyrosine Metabolism
0
1
2
3
4
5
6
7
Fold Enrichment
P value
1e-01
3e-01
4e-01
